# Activity of Cathelicidin Peptides against *Simkania negevensis*

**DOI:** 10.1155/2011/708710

**Published:** 2011-04-05

**Authors:** Manuela Donati, Antonietta Di Francesco, Maria Di Paolo, Natascia Fiani, Monica Benincasa, Renato Gennaro, Paola Nardini, Claudio Foschi, Roberto Cevenini

**Affiliations:** ^1^Section of Microbiology DESOS, Policlinico S. Orsola, University of Bologna, 40138 Bologna, Italy; ^2^Department of Veterinary Medical Sciences, University of Bologna, 40064 Ozzano dell'Emilia, Italy; ^3^Department of Life Sciences, University of Trieste, 34127, Trieste, Italy

## Abstract

The *in vitro* activity of six cathelicidin peptides against the reference strain Z of *Simkania negevensis* was investigated. Five peptides—PG-1, Bac7, SMAP-29, BMAP-27, and BMAP-28—proved to be active at very low concentrations (1 to 0.1 *μ*g/mL), while LL-37 cathelicidin was ineffective even at a concentration of 100 *μ*g/mL. In comparison to chlamydiae, *S. negevensis* proved to be more susceptible to the antimicrobial peptides tested.

## 1. Introduction

*Simkania negevensis* is an obligate intracellular Gram-negative bacterium belonging to the family of *Simkaniaceae* in the order *Chlamydiales* [1], discovered as a contaminant in a variety of cell cultures [2], and is able to grow also in various environmental free-living amoebae such as *Acanthamoeba poliphaga* [3].

Epidemiologic studies have reported a human widespread exposure to this bacterium [4, 5], both in healthy subjects and in association with respiratory diseases in infants and adults [6–10]. Coinfections with other pathogens have been described, such as respiratory syncytial virus in children and influenza virus and other bacterial species in adults [7, 8, 11]. A possible association between *S. negevensis* and acute rejection in lung transplant recipients has been suggested [12]. *S. negevensis* DNA has also been amplified from an aortic aneurysm [13, 14].

Previous investigations reported *S. negevensis in vitro* susceptibility to azithromycin, minocycline, erithromycin, doxycycline, and ofloxacin like chlamydiae [15] and the lack of susceptibility to ampicillin, penicillin G, bacitracin, cyclosporine, and fluoroquinolones [2, 15, 16].

Several studies [17–20] demonstrated the *in vitro* antichlamydial activity of peptides, such as cathelicidins, stored by mammalian leucocytes and show an antimicrobial activity against bacteria, fungi, protozoa, and enveloped viruses. With regard to the mode of action of these peptides against chlamydiae, previous investigations on the activity of protegrins [21] suggested an initial binding to lipopolysaccharide followed by insertion into the membrane that results in permeabilization. Yasin et al. [22] demonstrated that the antichlamydial activity of protegrins resided especially in residues 5 to 15 of native protegrin-1 and required both intramolecular disulfide bonds. 

In the present study, we investigate the *in vitro* activity of six antimicrobial peptides against the reference strain Z of *S. negevensis*.

## 2. Materials and Methods

The cathelicidin peptides—PG-1 from pig, Bac7 (1–35) from cattle, SMAP-29 from sheep, LL-37 from human, BMAP-27, and BMAP-28 from cattle—were chemically synthesized, purified, characterized, and provided as lyophilized peptide, as previously reported [19]. The reference strain Z of *S. negevensis* (American Type Culture Collection VR-1471) was grown in LLC-MK2 cells [23] in 6-large well plates and elementary bodies (EBs) were purified by use of sucrose gradients [24], resuspended in sucrose-phosphate-glutamic acid buffer (SPG) pH 7.4 (75 g sucrose, 87 mL 0.2 M Na_2_HPO_4_, 13 mL 0.2 M NaH_2_PO_4_, 0.72 g L-glutamic acid), and frozen in aliquots at −80°C. 

Stock solutions of each peptide at 1 g/liter were prepared in phosphate-buffered saline (PBS), pH 7.4, and stored frozen in 20-*μ*L aliquots at −80°C until used. To determine the lowest peptide concentration required to achieve ≥50% reduction of inclusions with respect to untreated control, the various peptides were diluted tenfold from 200 to 0.02 *μ*g/mL, in a volume of 100 *μ*L with PBS in polypropylene tubes. An equal volume of 5 × 10^5^ inclusion-forming units (IFU)/mL of purified *S. negevensis* EBs in SPG medium was then added, so obtaining a final cathelicidin concentration ranging from 100 to 0.01 *μ*g/mL in a mixture of PBS and SPG at a final pH 7.4. EBs untreated with cathelicidins were used as a control. After incubation at room temperature for 2 h, a 200 *μ*L aliquot was added to 800 *μ*L chlamydial growth medium and inoculated onto LLC-MK2 cells grown in 24-well plates in a 5% CO_2_ atmosphere. After centrifugation at 800 × g for 1 h at 33°C and incubation at 35°C for 72 h, the cultures were fixed and stained for the presence of inclusions by immunofluorescence using an in-house rabbit polyclonal hyperimmune serum raised against *S. negevensis*. The number of IFU per coverslip was counted in 40 microscopic fields using a ZEISS UV microscope at a magnification of ×200.

All tests were performed in triplicate and in three independent experiments. 

## 3. Results and Discussion

**Figure 1:**
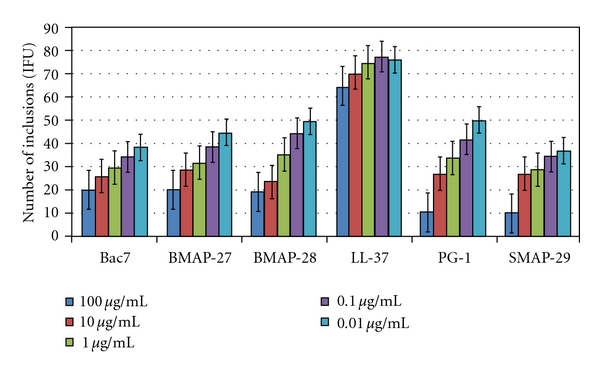
Activity of cathelicidin peptides at different concentrations on the infectivity of *Simkania negevensis. *

The activities of the six cathelicidin peptides against *S. negevensis* are reported in Figure 1 and are the means of results from the three experiments. In comparison with the infectivity of the untreated control (mean value 74 IFU/mL), Bac7 and SMAP-29 reduced by >50% (<37 IFU/mL) the inclusion numbers of *S. negevensis* at a concentration of 0.1 *μ*g/mL, whereas PG-1, BMAP-27 and BMAP-28 showed the same activity at a concentration of 1 *μ*g/mL. All these five cathelicidins demonstrated a very slow decrease in their inhibitory effect while their concentration has been reducing from 100 to 1 or 0.1 *μ*g/mL. LL-37 peptide did not exert any inhibitory effect, even at a concentration of 100 *μ*g/mL. 

In our previous study [19], the activity of six cathelicidins—PG-1, Bac7, SMAP-29, BMAP-27, LL-37 and BMAP-28—tested on chlamydiae of human and animal origin showed that five of them had inhibitory effect on *Chlamydia trachomatis* strains, SMAP-29 being the most active peptide at a concentration of 10 *μ*g/mL; on the contrary, LL-37 peptide was ineffective even at a concentration of 80 *μ*g/mL. With regard to the other chlamydial species tested, only *Chlamydophila pneumoniae* and *Chlamydophila felis* were sensitive to 10 *μ*g/mL of SMAP-29 and 80 *μ*g/mL of SMAP-29/Bac7, respectively.

In the present investigation, the same six cathelicidins were tested against the reference strain Z of *S. negevensis*. In comparison with chlamydiae, *S. negevensis* was not sensitive to LL-37 even at the concentration of 80 *μ*g/mL, but very sensitive to all the other five cathelicidins that showed an inhibitory effect at very low concentrations from 1 to 0.1 *μ*g/mL. To our knowledge no study on *S. negevensis* susceptibility to cathelicidins has been performed, until now. These preliminary results suggest a higher sensitivity of *S. negevensis* to cathelicidins respect to chlamydiae. A previous study showed differences in the susceptibility of different *C. trachomatis* serovars to antimicrobial peptides, suggesting that limited variations in the disulfide bonding in the membrane structure of these organisms may influence their susceptibility to peptides [18]. The membrane structure of *S. negevensis* probably differs significantly from that of other members of *Chlamydiales,* as monoclonal antibodies recognizing family-specific epitopes of chlamydial OMP-2, MOMP, and LPS did not bind *S. negevensis* antigens [2, 25]; in addition, only a relatively small number of common epitopes were shown to be shared when *S. negevensis* and other *Chlamydiales* members were tested for cross-reactivity by Western blot using polyclonal antisera [26]. The higher susceptibility of *S. negevensis* to cathelicidins in comparison to chlamydiae could be referred, to a great extent, to its different polypeptides pattern.
